# Fluoride Exposure through Different Drinking Water Sources in a Contaminated Basin in Guanajuato, Mexico: A Deterministic Human Health Risk Assessment

**DOI:** 10.3390/ijerph182111490

**Published:** 2021-10-31

**Authors:** Paulina Farías, Jesús Alejandro Estevez-García, Erika Noelia Onofre-Pardo, María Luisa Pérez-Humara, Elodia Rojas-Lima, Urinda Álamo-Hernández, Diana Olivia Rocha-Amador

**Affiliations:** 1 Instituto Nacional de Salud Pública, Universidad No. 655, Colonia Santa María Ahuacatitlán, Cuernavaca 62100, Mexico; jaestevezg@unal.edu.co (J.A.E.-G.); noeliaonofre@gmail.com (E.N.O.-P.); wicha.perez4@gmail.com (M.L.P.-H.); elos_15@hotmail.com (E.R.-L.); ualamo@insp.mx (U.Á.-H.); 2 Departamento de Farmacia, División de Ciencias Naturales y Exactas, Universidad de Guanajuato, Guanajuato 36050, Mexico; olivia2000_mx@hotmail.com

**Keywords:** fluoride, fluorosis, geogenic, groundwater, aquifer, risk assessment, Guanajuato, Mexico

## Abstract

Water fluoride levels above the World Health Organization’s guideline (1.5 mg/L), common in overexploited aquifers, represent a health hazard. Our objective was to assess the health risks posed by exposure to fluoride in different drinking water sources in a contaminated basin in Mexico. Fluoride was measured in mutual drinking water sources and in the urine of 39 children and women. Risks were estimated through hazard quotient (HQ) by drinking water source. Dental fluorosis was assessed in the children. Mean fluoride water concentrations (mg/L) were: well, 4.2; waterhole, 2.7; bottled, 2.1; rainwater, 0.4. The mean urinary fluoride concentrations (specific gravity adjusted) were 2.1 mg/L and 3.2 mg/L in children and women, respectively. Our multiple linear regression model showed children’s urinary fluoride concentrations increased 0.96 mg/L for every 1 mg/L increase in water fluoride (*p* < 0.001). Dental fluorosis was diagnosed in 82% of the children, and their HQ according to drinking water source was: well, 1.5; waterhole, 1.1; bottled, 0.8; harvested rainwater, 0.3. The pervasive dental fluorosis indicates a toxic past fluoride exposure; urinary fluoride levels and HQs indicate high exposure and current health risks for most children. Drinking harvested rainwater will likely prevent most of the local fluoride exposure.

## 1. Introduction

Groundwater provides almost 50% of the drinking water and about 40% of the agriculture irrigation water around the world. However, groundwater can contain toxic concentrations of naturally occurring elements. Furthermore, the increase in water demands of an ever-growing agricultural industry has led to an overexploitation of many freshwater aquifers worldwide, especially in rural arid and semi-arid regions [1]. This anthropogenic overexploitation can result in increased toxic concentrations of elements of geogenic origin, such as fluoride (F^−^), by promoting rock weathering, increased pH, changes in temperature, and other geochemical processes [2,3].

Even though F^−^ can have beneficial effects by preventing caries, especially when applied topically [4], chronic ingestion of F^−^ in concentrations once considered safe, and even helpful, is now known to cause toxic effects, mainly in early life stages, since F^−^ can cross the placenta [5] and the incomplete blood–brain barrier [6,7,8]. Hence, the U.S. Public Health Service recommendation for F^−^ concentration in fluoridated water for the prevention of dental caries was reduced to 0.7 mg/L. Still, naturally occurring F^−^ in groundwater is probably responsible for over 200 million people from among 25 countries estimated to suffer from fluorosis [9]. Moreover, F^−^ exposure inhibits Na+, K+-ATPase activity, thus contributing to many outcomes associated to this essential enzyme [10]; F^−^ has been associated with several health effects, including endocrine disruption (thyroid), diabetes [5], kidney and liver function damage [11], hypertension and cardiovascular effects [12,13], and particularly neurotoxicity. There is strong evidence from epidemiologic studies (including studies in Mexican children [14,15], experimental studies, and toxicokinetic studies that exposure to fluoride in early development is associated to neurotoxicity, resulting in intellectual disability [16].

In the high basin of the Laja River, or Cuenca Alta del Río Laja (CARL) in Guanajuato, Mexico, also known as the Independence Basin, water tables have been falling and F^−^ concentrations have been rising [2,3]. The CARL, located in central Mexico, has a semiarid climate, an annual mean precipitation of 498 mm (mostly falling from June to September), and an annual mean evaporation of 1877 mm. This has led to an overexploitation of the aquifer, with approximately 90% of the underground water being used for a constantly increasing crop irrigation demand [17]. Groundwater concentrations of F^−^ in the CARL have been documented at more than ten times the drinking water guideline of the World Health Organization, 1.5 mg/L [2], potentially affecting the local population of approximately 744,000 people [18]. As the information on high concentrations of toxic elements in the CARL underground water started permeating, community leaders, scientists, health authorities, and non-profit organizations started acting to promote safe drinking water [19]. The CARL’s inhabitants have searched for safer drinking water options based on the information they receive from the aforementioned groups and their risk perception. In 2014–2015, the first rain harvesting systems were installed in the CARL. Since then, several other communal systems have been installed, especially in buildings with large roof catchment areas, such as the community center and schools.

Even though underground water F^−^ levels have been well characterized in the CARL, its population’s actual fluoride exposure and health risks have not been studied. There is only one published study conducted, in what is vaguely referred to as an endemic fluorosis area in the state of Guanajuato, that evaluated dental fluorosis and measured urinary F^−^ concentrations; it does not explore F^−^ sources and it lacks scientific rigor in determining exposure for the time period relevant to the health outcome [20], since dental fluorosis can only occur in early life when the teeth are forming [21], hence the need for a comprehensive human health risk assessment.

A human health risk assessment of toxic agents is a systematized process used to estimate the nature and probability of adverse health outcomes in people who may be exposed to these agents present in environmental media currently, in the past, or in the future [22]. The risk assessment paradigm involves a problem formulation (as performed above), hazard identification and characterization (in this case, F^−^), exposure assessment, and risk characterization. The exposure assessment evaluates the amount of the potentially hazardous agent that reaches the target population. Exposure can be measured directly (using biomarkers), estimated with models, and/or generalized from existing information. Risk is determined by the coexistence of a hazard and an exposure to it. By combining the information of the exposure and a guideline value (level at which no adverse effects are expected), health risks are estimated [23].

This study’s objective was to carry out a deterministic assessment of the exposure and the health risks of children and women in the CARL who are exposed to different concentrations of F^−^, depending on their drinking water source.

## 2. Materials and Methods

### 2.1. Study Site

The CARL is located in the northeast of the state of Guanajuato (geographic coordinates: 21°33′ N, longitude 101°31′ W in the northeast, and 20°46′ N, 100°11′ W in the southeast), in turn located in central Mexico. It expands over an area of 7017 km^2^ [18] comprising seven municipalities: San Miguel de Allende, Dolores Hidalgo, San Luis de la Paz, San Diego de la Unión, Doctor Mora, San José Iturbide, and San Felipe.

**Figure 1:**
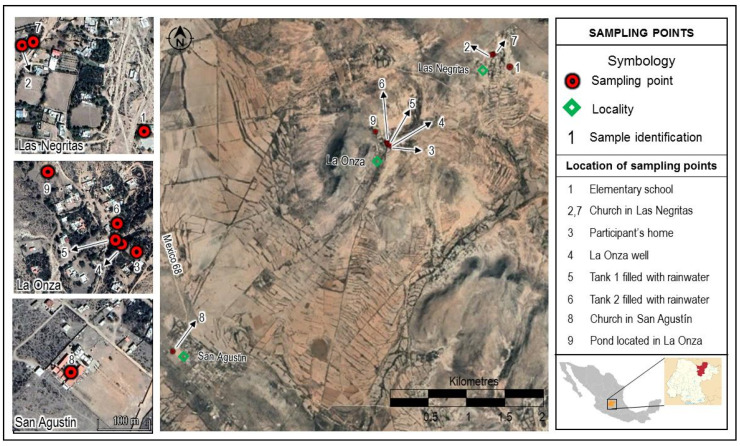
Study site in the high basin of the Laja River in Guanajuato, Mexico, and water sampling sites.

Within the CARL, the study site was selected in the San Luis de la Paz municipality, considering the presence of a well (La Onza) with documented past elevated F^−^ levels, a community center that we could use for working and lodging, and elementary schools with enough children to reach our population sample quota. As a result, we considered eight small villages that share two schools and churches: La Onza, Las Negritas, Vergel de Guadalupe, Puerto de Matancillas, Santa Rosa, Jaralillo, Encina, and Las Palomas; they are all rural areas inhabited by low-income families (Figure 1).

### 2.2. Study Population

Children 6–14 years old and their adult mothers or female guardians were invited to participate in the study. A meeting was convened for this purpose; it took place after Sunday mass in the patio outside the church, since most of the population is Catholic [18] and attends mass regularly. Besides this, the parish priest is a well-known and respected community leader that helped organize the recruitment.

We recruited the population sample (previously set at 40 children and 40 women) by convenience, due to time and budget restrictions.

Children of both sexes were eligible if they were between the ages of 6 and 14 years, were born and had always lived in the CARL, were in a mixed or permanent dentition stage, and their mother or guardian had agreed to both their own participation and their child’s.

Exclusion criteria for the children included having a sibling already enrolled in the study, having retained or missing teeth, or reporting a chronological age that did not match their teeth eruption timing.

Women were eligible if they were 18 years of age or older and agreed to their own participation as well as their child’s; they were not considered eligible if they had any physical or mental disorder that hindered their participation.

Both children and women were not considered eligible if they had a chronic kidney disease or current urinary infection, were receiving antibiotics or non-steroidal anti-inflammatory drugs, or had a history of treatment with tetracyclines.

### 2.3. Research Ethics

This study was conducted following the rules of the Declaration of Helsinki, and its protocol was approved by the Research Committee of the National Institute of Public Health, Mexico.

After explaining the study and answering all the questions about it, the women who agreed to participate signed an informed consent form for themselves and for their child, and each child who agreed to participate signed an informed assent form.

### 2.4. Dental Fluorosis Diagnosis

A single dentist carried out a visual clinical examination, consisting of direct observation with natural daylight and aided by intraoral mirrors to explore the permanent dentition. Each permanent maxillary incisor, molar, and mandibular molar was scored using Dean’s Index [24]. Canine, maxillary premolar, and mandibular premolar teeth were explored when at least 80% of the clinical crown was present.

The exploration results were registered in a specially designed registration card using Dean’s Index, according to the World Health Organization’s criteria [24]. The two most affected teeth were selected and, in case of discordant grades of fluorosis between them, the lowest grade was reported.

### 2.5. Anthropometric Measurements

A nutritionist weighed and measured the adult women and the children using a SECA^®^ 769 electronic column scale. She also measured twice the hip to waist ratio of each woman using a flexible measuring tape [25]. Using a stethoscope and a sphygmomanometer, a physician measured the women’s blood pressure in their upper arm, while they had been at least ten minutes at rest, were seated with a back support, had both feet on the ground, and their legs were not crossed [26].

### 2.6. Questionnaire

The participating women answered a questionnaire specially designed for this study to collect information on diet, lifestyle and health status of their family members, potential sources of exposure to F^−^, and risk perception. Drinking water sources were of special interest; the women were asked how much water, or beverages prepared with water, each family member drank per day and where that water came from (well, waterhole, bottled, or rain harvested). We also inquired about the water sources used for other activities, such as cooking and washing fruits and vegetables, which could potentially add to the water F^−^ exposure, depending on volume and F^−^ concentrations.

### 2.7. Water Samples

Duplicate water samples were obtained from all reported sources used for drinking: four different sites that received water from La Onza well (elementary school and church in Las Negritas, one participant’s home, directly at the well), four water tanks filled with stored rainwater (two from participants’ homes, Las Negritas and San Agustín churches), water from the waterhole located in La Onza (two samples collected directly from the waterhole at two different sites), and a purchased returnable 20 L water jug in one of the participating families’ home (Figure 1). Samples were collected in polyethylene containers with a screw-on lid with a capacity of 200 mL, previously rinsed with distilled water. The containers were filled to their maximum capacity, labeled, and stored at 4 °C in the dark until they were analyzed, according to the sanitary procedures for sampling water for human use and consumption in public and private water supply systems of the Mexican Official Norm NOM-014-SSA1-1993 [27].

### 2.8. Urine Samples

All participating women and children were asked for a first morning urine sample. An explanation was given on how to collect the sample and illustrated written instructions along with a sterile 120 mL polyethylene container with a screw-on lid were handed out to each participant. Containers were previously labeled with each participant’s identification number, and the information was checked when they were handed out.

Appointments were given to all participants at the local elementary school to turn in their samples. Upon receiving the sample, its identification number was checked to match the participant turning it in, the reception was recorded, and all the samples were kept in an ice box until they were transported to the laboratory. All samples were then stored at 4 °C until they were analyzed.

### 2.9. Specimen Analysis

The F^−^ concentration in water and urine was determined using the ion selective electrode method. We followed Protocol 8308 of the National Institute of Occupational Safety and Health (NIOSH) for fluoride in urine [28] and Protocol 9214 of the United States Environmental Protection Agency (US EPA) for fluoride in water [29]. Both methods are similar in terms of the calibration curve. Urine specimens were previously treated with 0.2 g of EDTA per 100 mL. A calibration curve was generated from 0.1 to 10.0 mg/L of F^−^. Standards and samples were mixed 1:1 with a total ionic strength adjustment buffer (TISAB I and II for urine and water, respectively). TISAB adjusts ionic strength, buffers pH to 5–5.5, and contains a chelating agent to break up metal–fluoride complexes. The equipment used for the potentiometric determination was a portable Orion 4-Star pH-ISE (Thermo Fisher Scientific, Waltham, MA, USA) by Thermo Scientific. The sample’s F^−^ concentration was determined by interpolation of the potential in the calibration curve. To confirm that the analytical procedure was under control, we reviewed records of millivolt reading for equivalent concentrations of F^−^ and the slope and linear regression values of the relationship of the mV readings to the concentrations of fluoride (slope between −58 ± 1 mV and r^2^ values > 0.99). The lower limit of detection (LOD) was of 0.10 mg/L. Urine and water samples were analyzed in duplicate and the mean F^−^ concentration was estimated for each sample. We used the IRIS Tech ClinChek^®^ Urine Control (RECIPE Chemicals + Instruments GmbH, Munich, Germany) lyophilised to trace elements in urine samples for quality control and found a relative accuracy of 96 ± 2%. For water samples, we prepared blind quality control standards (with low and high concentrations: 0.3, 0.7, 2, and 5 mg/L). The accuracy was 98 ± 3%. The quality control standards for urine and water were inserted at the beginning and for every 10 samples in each run.

In order to correct the F^−^ concentration by the urine dilution, specific gravity was measured with a hand-held ATAGO^®^ refractometer (Atago Ltd., Tokyo, Japan) with a range of 1.00–1.050 ± 0.001 U.G. Urinary fluoride concentrations adjusted by specific gravity were calculated for each participant through the following formula: UF_specific gravity_ = UF * (1.012 − 1)/(specific gravity − 1). A value of 1.012 is the median specific gravity of the study group. Specific gravity is less affected by body size, age, gender, and season than creatinine levels used for correcting dilution [30]. 

### 2.10. Statistical Analysis

A descriptive analysis of the data was carried out by estimating absolute and relative frequencies, central tendency, and dispersion measures. According to the variable type and distribution, means or proportions were compared in a stratified analysis by drinking water source (well, waterhole, bottled, or rainwater). To evaluate the association between urinary F^−^ concentrations adjusted by specific gravity as a dependent variable and the water F^−^ concentrations as an independent variable, a multiple linear regression model was run for children on the one hand and for the women on the other, adjusting by the village of origin, drinking water source, weight or body mass index (BMI), age, and sex of participants.

All the previous data were registered in an Excel file and exported and analyzed using Stata 13 software (StataCorp, College Station, TX, USA) [31].

### 2.11. Lifetime Average Daily Dose and Hazard Quotient

There are different approaches to estimating exposures. In this study, we used the scenario evaluation approach to estimate the average exposure of subpopulations of interest (estimating lifetime average daily dose), and the dose reconstruction (using urinary F^−^) approach to estimate each study subject’s individual exposure. The scenario evaluation approach, or indirect approach, combines information on a chemical’s environmental concentration with frequency and duration of exposure, as well as on the behaviors and characteristics of the exposed life stage; the exposure thus estimated is expressed in the same dose units as the drinking water reference dose for F^−^, thus allowing us to compare them. The dose reconstruction, or direct approach, estimates exposure through the measurement of biomarkers of exposure [32].

The lifetime average daily dose (LADD) of the study population was estimated by each water source currently used, for the children on the one hand and the women on the other. A daily water intake throughout their whole life was assumed. Water intake rate and body weight values were obtained from the mean results of the current study of the local population.

Current water F^−^ concentrations were estimated as the mean value of all the measurements of the same drinking water source. Past well water F^−^ concentrations were estimated with those reported by Caminos de Agua, a non-profit organization working with national and international universities and institutions to monitor groundwater quality, among other things; the concentrations considered for 2013, 2014, and 2017 were 2.76, 3.12, and 2.27 mg/L, respectively [33]. Since rain harvesting began in the study area four years prior to this study’s onset, all previous drinking water F^−^ concentrations were assumed to be represented by the 2013 well water result, which is the first one reported. For the year 2018, we used our field work well water measurement.

The following formula was used to estimate the lifetime average daily dose:

(1) $$LADD=\frac{C*IR*EF*ED}{BW*AT}\;$$

where:

*C* = water F^−^concentration (mg/L)
*IR* = mean per capita water intake rate (L/day)
*EF* = exposure frequency (days/year)
*ED* = exposure duration (years)
*BW* = body weight (kg)
*AT* = averaging time (days)

A hazard quotient (*HQ*) is used to estimate noncancer health risks; it is the ratio of the potential exposure to a substance (in this case *LADD*) and the level at which no adverse effects are expected (reference dose). A hazard quotient of 1 or lower means noncancer health effects are unlikely and can thus be considered to have negligible hazard. For HQs greater than 1, potential adverse health effects can be expected. However, HQs do not indicate statistical probabilities of adverse effects occurring, they only indicate whether and by how much the exposure dose exceeds the reference dose [34]

The hazard quotient was estimated with the following formula:

(2) $$HQ=\frac{LADD\;}{RFD}\;$$

where:

*RFD* = reference dose (mg/kg/day), as reported by the United States Environmental Protection Agency’s (EPA) Integrated Risk Information System = 6 × 10^−2^ mg/kg-day [35], defined as “an estimate (with uncertainty spanning perhaps an order of magnitude) of a daily oral exposure to the human population (including sensitive subgroups) that is likely to be without an appreciable risk of deleterious effects during a lifetime. It can be derived from a no observed adverse effect level (NOAEL), lowest observed adverse effect level (LOAEL), or benchmark dose, with uncertainty factors generally applied to reflect limitations of the data used” [36]; in this case, it is derived from an epidemiologic study in children [36].

## 3. Results

The final sample size consisted of 39 woman–child pairs, since one pair had to be eliminated for not turning in a urine sample. The families of the study participants were composed of 3 to 10 members, with a mean of four.

On average, the women were almost 36 years old and had lived 95% of their lives in the study area; none had more than nine years of formal education and none reported smoking. The children’s ages ranged from 6 to 14 years with a mean of 9.5; they had all lived their whole lives in the studied area.

Staple foods of our study population were legumes (mostly beans), corn-flour tortillas, cereals, pasta, bread, potatoes, chili peppers, nopales, carrots, prickly pear fruit, chicken, and eggs.

When asked if their children had dental fluorosis, only 25% of the women reported that they had it. However, the dentist diagnosed some level of dental fluorosis in 82% of the children.

The main characteristics of the study population, both women and children, are described in Table 1.

**Figure 2:**
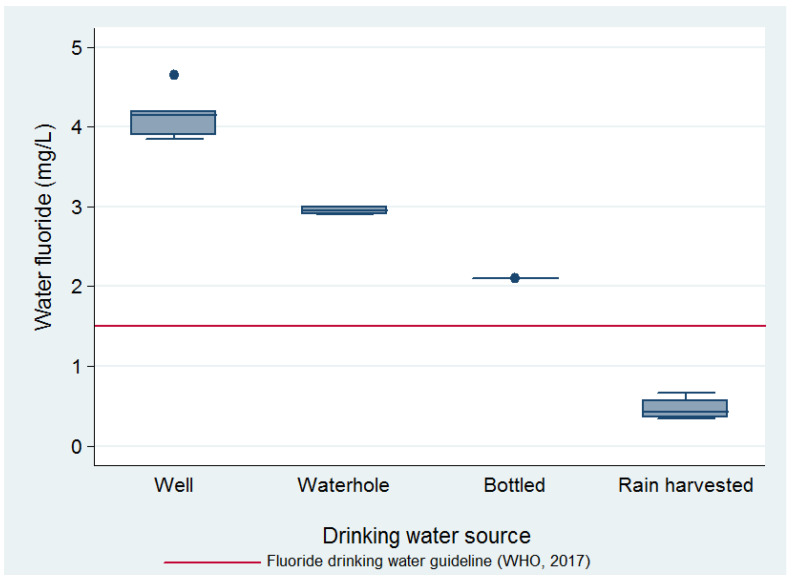
Water fluoride mean concentrations by drinking water source and in relation to the World Health Organization’s (WHO) drinking water guideline.

Water F^−^ concentration (mg/L) ranges per source used for drinking were: well, 3.7–4.9; waterhole, 2.9–3.0; bottle (jug), 2.1; rain-harvest, 0.37–0.71. The mean F^−^ concentrations of the water samples by source, as well as their relation to the World Health Organization’s (WHO) guideline value for drinking water of 1.5 mg/L, can be seen in Figure 2.

Drinking water sources were reported by the women for their whole family; depending on the source of this drinking water, they carried out either none or different options of water treatment to deem it fit for drinking. The drinking water source used by each family was mostly split between rain-harvested and bottled, while only 2% of participants used waterhole water and 13% used well water for drinking. Since the waterhole fills from underground water and rain, its fluoride levels were the second highest after the well’s, and only 2% of the participants used it for drinking, this source was lumped with the well water drinking source in subsequent analyses.

The summary of the water sources and their corresponding treatments reported by the women can be seen in Table 2.

The children’s urinary F^−^ levels ranged from 0.51 to 7.5 mg/L, with a mean (SD) of 2.1 (1.8) mg/L; the women’s urinary F^−^ levels ranged from 0.78 to 13.9 mg/L, with a mean (SD) of 3.1 (2.2) mg/L. The women’s mean urinary F^−^ levels were significantly higher than the children’s when compared by T-test (*p* value = 0.02). There was also a significant difference in the children’s and women’s mean urinary F^−^ level, depending on the drinking water source used, as shown in Table 3. Furthermore, the difference in the mean urinary F^−^ adjusted by specific gravity of children who drank well or waterhole water and children who drank bottled or rain-harvested water is 3.32 mg/L higher in the former group (95% CI 1.81–4.82), *p* < 0.001.

**Figure 3:**
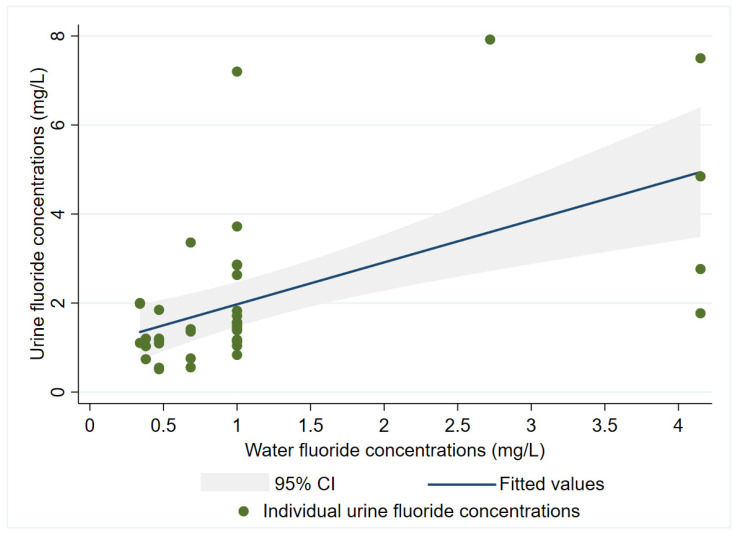
Urinary fluoride concentrations predicted by water fluoride concentrations and adjusted by age, sex, and BMI in 6–14-year-old children.

A multiple linear regression model adjusted by age, sex, and BMI showed that the children’s urinary F^−^ concentrations were predicted by water F^−^ concentration with a β = 0.96 (95% CI 0.46–1.45), *p* < 0.001, and an adjusted r^2^ = 0.33. Age, sex, and BMI were not statistically significant (*p* > 0.05), and their coefficients were −0.04 (years), −0.26 (reference = female), and 0.06, respectively (Figure 3). As for the women’s model, urinary F^−^ concentrations were predicted by water F^−^ concentration with a β = 0.58 (95% CI −0.06–1.12), *p* = 0.054, and an adjusted r^2^ = 0.07. Age was not statistically significant and had a β = 0.03 (years), and BMI categories showed no significant differences either (*p* > 0.05).

Regarding the estimates of the dose delivered at the exposure point, considering the volume used in the water used for cooking or washing fruits and vegetables, the amount of F^−^ this would potentially add to the total F^−^ exposure dose would be negligible and was therefore not taken into account in the LADD. The US EPA Exposure Handbook does not account for water used for washing fruits and vegetables [33], and we found no other source that does. Moreover, adding a few drops (each 0.05 mL) of well water (the one with the highest F^−^ levels) that might have remained on fruits and vegetables, and thus ingested, to the mean volume of water drank (>1000 mL/day), represented an infinitesimal amount of F^−^.

The HQs estimated for the scenarios of drinking water from the waterhole or well in the CARL were greater than the acceptable value of <1 for both children and women, whereas those for drinking bottled or rain-harvested water were not. The values of the F^−^ LADDs and the corresponding HQs for each drinking water source and by the studied subpopulations of women and children are shown on Table 3.

## 4. Discussion

This study’s different results suggest past, present, and potentially future exposure to F^−^ levels above the international drinking water guidelines, and that consequently represents a health risk for children and women whose drinking water source is the La Onza well or wells with similar F- concentrations in the CARL. Since convenience sampling is non-probabilistic, it cannot pretend to be representative of the whole population. However, we have no reasons to believe (although we have no evidence) those who were recruited in this very homogenous population were different in terms of drinking water volume or body composition from those who were not recruited.

A dental fluorosis prevalence above 80% in the 6–14-year-old children from CARL is an unequivocal sign of a pervasive early life and/or prenatal (past) F^−^ exposure; that it is also a sign of toxicity is debatable, since some argue that dental fluorosis may be a cosmetic rather than a toxic effect. However, IQ reductions were seen in a meta-analysis of water F^−^ exposure even at levels = 1 mg/L [37], which corresponds to the NOAEL for objectionable dental fluorosis (from mild to severe) [35] seen in 37% of this study’s children. Moreover, IQ reductions were seen in children prenatally exposed to low F^−^ levels: an increase of 0.5 mg/L in maternal urinary F- predicted a lower IQ by 2.5 points in the offspring (95% CI −4.12, −0.59); mean maternal pregnancy urinary F^−^ levels in those 299 pregnant women from Mexico (0.90 mg/L) were three times below what we found in this study [15]. If the previous association holds true for our population (assuming the women had similar urinary F^−^ levels when they were pregnant = 3.1 mg/L), we could expect children from our study area now to have a 10-point reduction (2.4 to 16.5) from their potential IQ.

Additionally, a study in China of children of similar age (7–13 years old) to ours found that in children with urinary F- concentrations > 1.7 mg/L, every increase in 1 mg/L above that level was associated to a reduction of almost five potential IQ points (95% CI: −9.198, −0.732, *p* = 0.022); since our children’s mean urinary F^−^ concentrations were >1.7 mg/L, we could probably expect similar results. Children in the aforementioned study who were prenatally exposed fared worse than those exposed after birth, but no increased effect was seen in children exposed both prenatally and afterwards [38] Since 95 % of the women and 100% of the children in our study had lived all their lives in the study area, we can assume a prenatal plus a continuous postnatal exposure to F^−^.

Even more so, a quantitative risk analysis aimed at finding a safe daily F^−^ dose for children found that to protect against a five-point IQ loss, this dose would possibly be 0.045 mg F^−^/day; children in our study drinking a mean volume of approximately 1 L of water/day with a F^−^ concentration of 4.2 mg/L, if it comes from the local well, are ingesting more than eight times this F^−^ dose [39].

Given our result that for every 1 mg/L increase in water F^−^, the urinary concentration of the studied children increases by 0.96 mg/L, it is probably safe to assume that the source of drinking water plays a major role in determining these children’s exposure to F^−^ and its potential health effects. In a cohort of Canadian children, water F^−^ concentrations (mean = 0.35 mg /L) were significantly associated to urinary F^−^ concentrations in a model controlling for age and sex, just as we carried out, but the magnitude of the association was much lower (β = 0.44, 95% CI: 0.30, 0.59, *p* < 0.001) [40].

Harvested rainwater was the only water source below the World Health Organization’s F^−^ drinking water guideline, but it was not fluoride-free. Measurements of rainwater F^−^ concentrations can vary considerably in one place depending on weather conditions or in different places depending on local sources. In Nigeria, rainwater F^−^ concentrations were 0.02 mg/L during the southeast monsoon and 0.1 mg/L during the northeast monsoon [41]. The existence of nearby sources of airborne fluoride emissions has been seen to increase the rainwater F^−^ concentrations: in Vanuatu, a volcano produced F^−^ rainwater concentrations ranging from 0.7 to 9.5 mg/L [38], and in Wielkopolski National Park in Poland, located 10 km away from chemical plant that emits fluoride, F^−^ rainwater concentrations ranged from 0.32 to 0.55 mg/L [41]. Our hypothesis is that in the CARL, a semi-arid, dusty, and windy area, groundwater containing high levels of F^−^ that is used for irrigation will evaporate and might leave behind F^−^ contaminated soil and dust; these dust particles may in turn contaminate rainwater while it falls or once it is harvested, stored, and gradually used for months during the dry season.

Even though we only measured one sample of bottled water, F^−^ levels were similar to those found in bottled water samples from two other states of Mexico with endemic fluorosis, Durango and Jalisco (2.0 and 2.9 mg/L) [15] Bottled water F^−^ levels from non-fluorosis endemic areas, such as Mexico City, have found inconsistent results, sometimes being below the Mexican norm [42] and sometimes having samples above the norm. It is imperative to have representative and sufficient F^−^ level measurements in bottled water in the CARL, since it seems to be an inefficient measure in avoiding water F^−^ exposure; the users are incurring a costly measure for a source that is still above the optimal F^−^ concentrations.

The F^−^ concentrations in the waterhole are consistent with what would be expected, since rainwater dilutes the underground water that fills it. However, it will be important to inform the population of its high F^−^ contents, given that it perceives it as a very safe, although not easily accessible, drinking water option.

A present F^−^ health risk to the CARL population that drinks well water with F^−^ concentrations that were approximately three times the WHO’s F^−^ drinking water guideline, especially for the children, is suggested by the HQs above 1, reflecting a mean exposure above the reference dose [43]. Additional evidence suggesting potential current F^−^ health risks is the children’s exposure reflected in mean urinary F^−^ concentrations, which for those who drink well water are ten-fold the urinary F^−^ mean of the Canadian children who presented an association, albeit weaker than at younger ages, with performance IQ (B = −1.51, 95% CI: −2.90, −0.12) [44].

Apart from contaminated water, only soda seems to be another potentially significant source of fluoride intake in the CARL, where risk perception of contaminated drinking water has led to an increased consumption of soda, based on the belief that it is free from toxic elements such as F^−^, but this has proved erroneous, as previous studies in the state of Guanajuato [45] and Mexico [42,46] have found. It is paramount to carry out a quantitative F^−^ assessment to inform the population in this regard, because drinking soda not only has higher F^−^ levels than water but also implies other health risks.

Salt in Mexico is fluoridated in an attempt to prevent dental caries, but exceptions to the Mexican Official Norm mandating this are observed in places where water fluoride concentrations are naturally above 0.7 mg/L [47]; the whole state of Guanajuato is exempt from the distribution of fluoridated salt [48]. We did not apply a detailed food frequency questionnaire in this study, but we inquired about the weekly consumption of groups of foods and we were fed by community volunteers during our filed work; we can conclude that the studied population’s diet is not high in the main food group sources of F^−^, especially in Mexico, according to a study that included the most representative foods and beverages consumed at the national level: seafood, meats and poultry, or fast food [46].

Even though 82% of the children had a professional diagnosis of some level of dental fluorosis, this was only perceived in 25% of the children by their mothers or guardians. This is probably explained by the fact that in 45% of the cases, the children presented a very mild fluorosis (corresponding to opaque, paper-white areas involving less than one fourth of the tooth surface), which might be missed by non-experts or may not even stand out as something altered due to its high prevalence. On the other hand, the data suggest that awareness of dental health problems has probably led to a change in the drinking water source: 100% of those who now drink bottled water had dental fluorosis, whereas only 60% of those who drink well water had dental fluorosis.

Future F^−^ health risks are to be expected in the CARL, since 15% of the families studied still drink well water. Besides, 66% of those who still drink well water give it no treatment to reduce F^−^. The investigated water treatment methods do not necessarily reduce water F^−^, but the fact that they use none probably reflects a lack of risk perception or information. Finally, groundwater F^−^ levels are expected to increase because of deeper drilling of wells that reach older water that is richer in F^−^, more urban pumping that upwells mineralized groundwater, and less rainfall recharge along with more diverse and F^−^ contaminated recharge waters [3].

Even though the groundwater geogenic contamination problem has been studied from the hydrogeology [2,3], regulatory [49], and dental [20] perspective in the CARL, the human exposure and health risk magnitude have not been investigated in-depth. With this study, we were able to produce important diagnostic results that reduce the assumptions and increase the precision of the exposure assessment. For instance, the CARL being a semi-arid region, 6–14-year-old local children reportedly drink approximately three times more water than the mean assumed by the EPA for their age group. Still, this study was limited by the small sample size and the few drinking water samples; future studies will benefit from increasing their numbers and including follow-up measurements.

## 5. Conclusions

This is the first study to analyze the association between F^−^ concentrations in different drinking water sources and urinary F^−^ concentrations in children and women in the CARL. We observed a strong association in magnitude and significance of both the water F- levels prediction of urinary F- levels in children (β = 0.96 and *p* value < 0.001) and of the difference in urinary F- levels depending on drinking water source in women and children (*p* value < 0.05). Rain-harvested water proved the only source below the F^−^ WHO drinking water guideline, and children who reportedly drank this water were the only ones with mean urinary F^−^ levels (1.3 mg/L) below those associated with neurotoxicity in children exposed in different windows of early life (1.7 mg/L). It is also the first study to identify a non-probabilistic health risk of the population of women, and especially children, associated with well water F^−^ concentrations, since the HQs of this source (1.25 and 1.5, respectively) indicate an exposure above the reference dose.

Even though our sample size was small and recruited by convenience (not guaranteeing representativeness), the results of this risk assessment are especially relevant to other CARL populations. On the one hand, we might be able to extrapolate the exposure dose and health risks to other inhabitants who share sociodemographic characteristics and drink water from the several existing wells with similar or higher F^−^ concentrations than the well we studied. On the other hand, our results are important given a possible increase of aquifer F^−^ concentrations in the future, as the trends suggest in some local wells, due to increased overexploitation in response to population growth and agricultural water demand expansion.

In spite of our study’s small population sample and number of sampled water sources, which will need to be built upon in future studies to decrease uncertainty, our results strongly suggest that exposure to toxic drinking water F^−^ levels is preventable in the CARL by switching to harvested rainwater. Future studies could complement these results by quantitatively assessing F^−^ exposure through diet, especially considering soda and confirming that local salt is indeed not fluoridated.

## Figures and Tables

**Table 1:** General characteristics of the studied women and children from the High Basin of the Laja River.

Adult Women *N* = 39		Mean (SD)	Percentage
Age (years)		35.7 (7.9)	
**Education**	Less than six years		18
	Elementary		62
	Junior high		20
**Village of origin**	Puerto de Matancillas		38.4
	Las Negritas		25.5
	La Onza		15.4
	Jaralillo		10.3
	Encina		2.6
	Santa Rosa		2.6
	Las Palomas		2.6
	Vergel de Guadalupe		2.6
Water consumed per day (L)		1.63 (0.83)	
Soda consumed per day (L)		0.4 (0.26)	
Weight (kg)		68.3 (11.7)	
BMI (kg/m^2^)		28.8 (3.1)	
18.5–24.9			31
25.0–29.9			28
≥30			41
**Waist-to-hip ratio**		0.87 (0.05)	
Recommended (<0.8)			48
Increased health risk ≥ 0.80			62
**Systolic blood pressure (mm Hg)**		111.9 (21.0)	
**Dyastolic blood pressure (mm Hg)**		72.0(9.9)	
Children *N* = 39		Mean (SD)	Percentage
Age (years)		9.5 (2)	
**Sex**	Male		46
	Female		54
Water consumed per day (L)		1.02 (0.67)	
Soda consumed per day (L)		0.35 (0.25)	
Weight (kg)		35.1 (9.4)	
**Dental fluorosis** **(Dean’s Index)**	Normal		15.8
	Questionable		2.6
	Very mild		44.7
	Mild		26.3
	Moderate		5.3
	Severe		5.3

**Table 2:** Sources of water for different uses, their treatment, and fluoride concentrations in the High Basin of the Laja River, 2018.

Water Use (Percentage)		Water Source		
		Well or Waterhole	Bottle	Rain-Harvest
Drinking		15	41	44
Treatment of drinking water	Boiling	17	6	6
	Filtering	17	0	53
	Chlorinating	0	0	6
	None	66	94	35
Cooking		51	8	41
Washing fruits and vegetables		62	5	33

**Table 3:** Fluoride exposure and health risk assessed by urinary fluoride concentration, lifetime average daily dose (LADD), and hazard quotient (HQ) in 6–14-year-old children and women from the High Basin of the the Laja River.

Estimate			Drinking Water Source			
			Well	Waterhole	Bottled	Rain-Harvested
Exposure (Biomarker)	Urinary fluoride ^1^ (mg/L) Mean (±SD)	Children *	5.0 (2.8)		2.1 (1.5)	1.3 (0.7)
		Women **	4.5 (4.7)		3.2 (1.3)	2.4 (1.2)
Exposure (Population mean estimate)	LADD (mg/kg/day)	Children	0.0883	0.0648	0.0501	0.0180
		Women	0.0748	0.0619	0.0504	0.0180
Health risk	HQ ^2^	Children	1.5	1.1	0.8	0.3
		Women	1.2	1.0	0.8	0.3

* ANOVA test *p* value = 0.02, ANOVA test *p* value ** < 0.001, ^1^ adjusted by specific gravity, ^2^ fluoride reference dose (RFD) = 0.06 mg/kg/day [36].

## Data Availability

The data presented in this study are available on request from the corresponding author. The data are not publicly available due to privacy issues.
